# Development and Optimization of Epigallocatechin-3-Gallate (EGCG) Nano Phytosome Using Design of Experiment (DoE) and Their In Vivo Anti-Inflammatory Studies

**DOI:** 10.3390/molecules25225453

**Published:** 2020-11-20

**Authors:** Mohammad H. Shariare, Kazi Afnan, Faria Iqbal, Mohammad A. Altamimi, Syed Rizwan Ahamad, Mohammed S. Aldughaim, Fars K. Alanazi, Mohsin Kazi

**Affiliations:** 1 Department of Pharmaceutical Sciences, North South University, Dhaka 1229, Bangladesh; mohammad.shariare@northsouth.edu (M.H.S.); afnan.kazi@northsouth.edu (K.A.); Iqbal.faria@northsouth.edu (F.I.); 2 Department of Pharmaceutics, College of Pharmacy, King Saud University, Riyadh 11451, Saudi Arabia; maltamimi@ksu.edu.sa (M.A.A.); afars@ksu.edu.sa (F.K.A.); 3 Central Laboratory, Department of Pharmaceutical Chemistry, College of Pharmacy, King Saud University, Riyadh 11451, Saudi Arabia; srahamad@ksu.edu.sa; 4 Research Center, King Fahad Medical City, P.O. Box. 59046, Riyadh 11525, Saudi Arabia; maldughaim@kfmc.med.sa

**Keywords:** nanophytosome, epigallocatechin-3-gallate (EGCG), particle size, drug loading, anti-inflammatory

## Abstract

Inflammation is responsible for the development of many diseases that make up a significant cause of death. The purpose of the study was to develop a novel nanophytosomal preparation of epigallocatechin-3-gallate (EGCG) and egg phospholipid complex that has a lower particle size with higher drug loading capability, physical stability and anti-inflammatory activities. The impact of different factors and material characteristics on the average particle size was studied along with the drug loading of phytosome using design of experiment (DoE). The in vivo anti-inflammatory study was evaluated using a rat model to investigate the performance of EGCG nanophytosome. UHPLC results showed that 500 µg of EGCG were present in 1 mL of green tea extract. SEM data exhibited that phytosome (phospholipid-drug complex) was in the nanosize range, which was further evident from TEM data. Malvern Zetasizer data showed that the average particle size of the EGCG nanophytosome was in the range of 100–250 nm. High drug loading (up to 90%) was achieved with optimum addition rate, stirring temperature and phospholipid concentration. Stability study data suggest that no significant changes were observed in average particle size and drug loading of nanophytome. The in vivo anti-inflammatory study indicated a significant anti-inflammatory activity of green tea extract, pure EGCG and its phytosomal preparations (*p* ≤ 0.001) against acute paw edema.

## 1. Introduction

Inflammation is one of the major reasons behind many diseases [1,2]. In particular, chronic inflammatory diseases are a major cause of global mortality. Chronic disease is ranked as the highest risk to human health according to the World Health Organization (WHO). Globally, three out of every five individuals die due to chronic inflammatory diseases such as stroke, chronic respiratory diseases and heart disorders. Currently, around 2.1 million Americans suffer from rheumatoid arthritis [3]. Furthermore, people of Bangladesh suffer from chronic inflammation such as stroke (16.27%), coronary heart disease (14.31%), tuberculosis (8.77%), lung disease (8.69%) and diabetes mellitus (5.09%) (WHO, 2017). Non-steroidal anti-inflammatory drugs (NSAIDs) are the most commonly used drugs to treat patients’ inflammation. Aspirin is recognized as the first synthetic NSAID to be introduced in the market [4]. However, long-term use of these medications results in severe adverse effects such as gastric mucosal damage, rashes, angioedema and pruritus. There has been advancement in scientific investigations into the development of drugs with anti-inflammatory properties from medicinal plants owing to side effects associated with synthetic drugs [5].

Phytoconstituents such as polyphenols like flavonoids, terpenoids, tannins and xanthones are chemical compounds present in plants with therapeutic properties. Phytoconstituents (flavonoids, tannins, etc.) were found as a useful alternative to synthetic drugs for a long period. While phytoconstituents have potential effectiveness and safety profile, they exhibit poor absorption properties when given orally because of their reduced aqueous solubility, poor membrane permeability and rapid metabolism. Therefore, the bioavailability of these hydrophobic molecules is translated to reduced clinical application [6]. Polyphenols such as quercetin, resveratrol and tannins showed anti-inflammatory activities due to their strong free radical scavenging action. A previous study showed that resveratrol possesses anti-inflammatory activity [7]. Resveratrol showed anti-inflammatory properties in several other diseases like arthritis [8], pancreatitis [9] and experimental colitis [10]. 

*Camellia sinensis*, also commonly known as tea, is the most regularly consumed beverage worldwide [11,12]. Green tea exerts significant sound effects on human health [13]. Catechins, among different polyphenol constituents, are found in green tea extract. The main flavanols found in green tea are epigallocatechin-3-gallate (EGCG), epigallocatechin (EGC), epicatechin-3-gallate (ECG) and epicatechin (EC). EGCG is the major and potent green tea polyphenol, accounting for 50–75% of the total catechins [14]. EGCG inhibits several physiologically important anti-oxidant enzymes and shows excellent antioxidant activities [15]. EGCG has shown beneficial effects on diabetes, cardiovascular diseases, Parkinson’s disease, Alzheimer’s disease, stroke and obesity [12,16]. EGCG possesses anti-mutagenic and anti-carcinogenic effects of environmental agents that increase the risk of cancer development [17,18]. Green tea extract containing EGCG inhibited carcinogenesis in different animal models with lung, skin, esophagus and liver cancers [19,20]. In vivo studies showed that EGCG increases total plasma antioxidant activity and decreases plasma total cholesterol and blood triglyceride levels when given at high doses [21,22].

However, EGCG exhibits low bioavailability due to poor absorption across the gastrointestinal tract (GIT) [23]. Several nano-delivery systems can be used for EGCG, including solid lipid nanoparticles, liposomes, polymeric nanoparticles, gold nanoparticles, inorganic nanocarriers and protein/peptide-based nanocarriers. Lipid nanoparticles showed enhanced stability of EGCG to facilitate the release characteristics and bioavailability in vivo. Chitosan coated nano-lipid composites were synthesized to prevent atherosclerosis which improved stability and cell uptake of EGCG [24,25,26,27] and demonstrated that nano-preparation significantly improved the oral bioavailability of EGCG compared to free EGCG. It is reported that EGCG uptake when incorporated in a solid self-double emulsifying drug delivery system (SDEDDS) was much higher compared to the pure drug [28]. Liposomes synthesized with nonionic surfactants and cholesterol show improved oral absorption and stability of EGCG [29]. Chen et al. (2014) prepared nonradioactive gold nanocarriers coated with EGCG which showed marked cytotoxic effect in in vitro and in vivo studies [30,31]. It has been shown that oil in water (O/W) emulsions are promising delivery systems for efficient topical delivery of EGCG [32].

Phytosomes are little cell-like structures made when solids obtained from plant extract are dispersed in a dietary phospholipid matrix, known as lecithin. These are novel transportation techniques employed to improve the absorption and bioavailability of phytoconstituents due to the amphiphilic characteristics and emulsifying action of phospholipids. In contrast to liposomes, where the active ingredient in the core is surrounded by a micellar structure made of phospholipids, phytosomes contain the active ingredient attached to the polar head of the phospholipid, being an essential element of the micellar membrane. Active ingredients in herbal extracts showed better biological activity when provided in a phytosome formulation. Phytosomes also help to reduce the dose and increase the duration of action of herbal extracts’ active ingredients because of their sustained drug release pattern. A previous study suggests that phytosomal preparation of commercial green tea extract enhanced the absorption of catechins compared to free EGCG [33,34,35,36,37].

This work aimed to develop stable nanophytosome of EGCG with a low average size and high loading efficiency. It was also a main focused to identify critical process parameters and material attributes which affect the average size and drug loading of phytosome. The impact of different process parameters and material attributes on the average particle size and the drug loading of phytosome was evaluated using design of experiment (DoE). An in vivo anti-inflammatory study was also evaluated using a rat model to investigate the performance of nanophytosomal preparation of EGCG compared to green tea extract and pure EGCG.

## 2. Results and Discussion

### 2.1. Identification and Quantification of EGCG by UHPLC 

**Figure 1:**
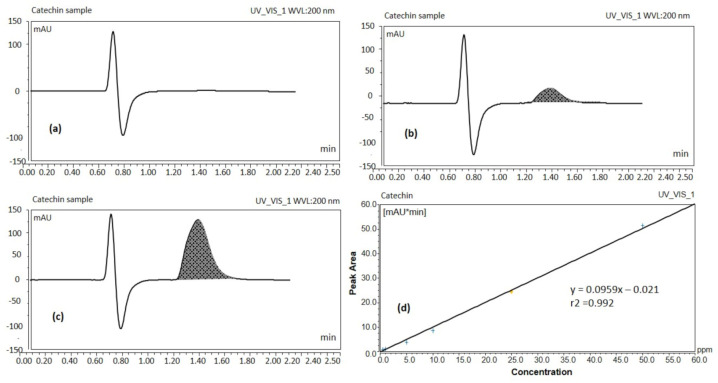
UHPLC chromatograms of blank sample (**a**), catechin standard solution 5 ppm (**b**), catechin of the real sample (green tea leaf extract solution) (**c**), UHPLC calibration curve of catechin (**d**) (UV detection at 200 nm).

UHPLC results showed the blank sample containing methanol only, (Figure 1a) and standard solution of catechin 5 ppm (Figure 1b) which suggests that the separation of the catechin peak and its detection was ideal without interference from other components present in tea extract. The catechin analyte was adequately separated at a retention time of ~1.617 min without any degradation product (Figure 1b) from the extracted real sample for the total run time of ~2.5 min. 

The peak response of catechin from the calibration curve was linear over the concentration range of 0.5 to 50 ppm. Peak areas of catechin were plotted against selected concentrations and linear regression analysis was performed on the resultant curve. The regression equation for the calibration curve was found to be *y = 0.0959x* – 0.021 with r^2^ value = 0.992 (Figure 1d). Where *y* denotes the peak area of the analyte catechin, *x* the concentration of the catechin and r^2^ the correlation coefficient. Results obtained from UHPLC analysis showed that 500 μg EGCG were present per mL of green tea extract. The proposed method has been used for routine analysis of the batches of catechin in the current studies.

### 2.2. Identification of EGCG by GC/MS 

**Figure 2:**
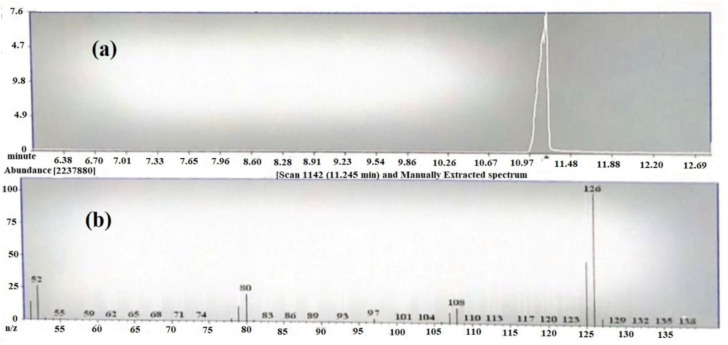
(**a**) TIC chromatogram of epigallocatechin-3-gallate (EGCG) by GC-MS and (**b**) mass spectrum of 1,2,3-benzenetriol.

The Gas chromatography/mass spectroscopy result at 20 eV showed a prominent peak at room temperature (RT) 9.6 min (Figure 2a). The peaks resulted from broken fragments of 1,2,3-benzenetriol from EGCG. The mass spectra showed the presence of a base peak at 126 of benzenetriol (Figure 2b). The peak of the benzenetriol was identified by comparing the spectra with that of the NIST 2005 (National Institute of Standard and Technology library) and Wiley 2006 library. The prominent peak at 126 and 80.1 showed the confirmation of 1,2,3-benzenetriol. The total run time was 16 min and the peak of benezenetriol was of good shape and completely resolved.

### 2.3. Scanning Electron Microscopy (SEM) of EGCG Phytosome

**Figure 3:**
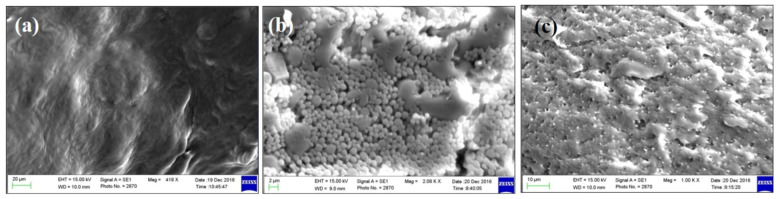
SEM micrographs of (**a**) phospholipid, (**b**) green tea extract and (**c**) green tea extract-loaded phytosome (batch 15).

Figure 3 shows the results from the SEM of green tea extract and the phospholipid–green tea extract complex (phytosome). Figure 3c shows that green tea extract containing EGCG was successfully loaded on phospholipid.

### 2.4. Transmission Electron Microscopy (TEM) of EGCG Phytosome

**Figure 4:**
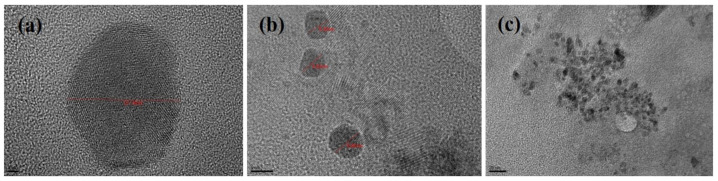
TEM micrographs of green tea extract-loaded nanophytosomes (batch 15) scales showing (**a**) 2 nm, (**b**) 5 nm and (**c**) 20 nm range.

The TEM images of phytosomal preparation of green tea extract showed particles with octagonal shapes within the nanometer size range as shown in Figure 4.

### 2.5. Particle Size Analysis of Phytosomal Batches of EGCG

**Figure 5:**
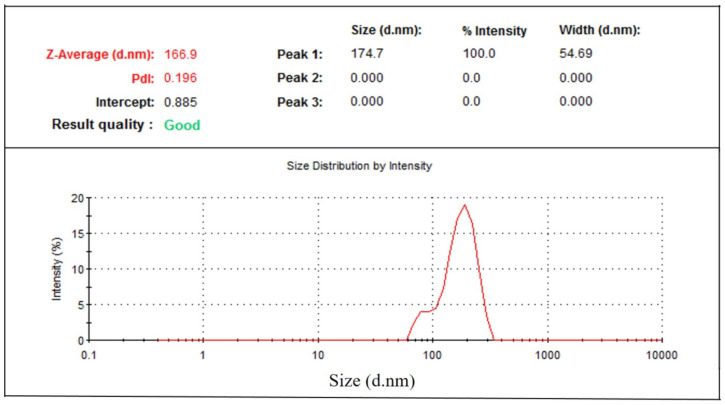
Particle size distribution of green tea extract-loaded nanophytosome.

Particle size distribution data showed that phytosomal batches of green tea extract were in the range of 100–250 nm (Table 1) when characterized using dynamic light scattering (DLS). Results (Table 1) show that phytosomal batch no. 15 has the lowest average particle size with high drug loading efficiency. Figure 5 shows an example of the particle size distribution results of the nanophytosomal batches, in which the sample produced a low PDI value. It could be stated that the lowest PDI value is close to the mono-disperse state of the nanoparticles.

### 2.6. Impact of Processing Parameters on Average Particle Size and Drug Loading of Phytosomal Batches of Green tea Extract Containing EGCG

**Figure 6:**
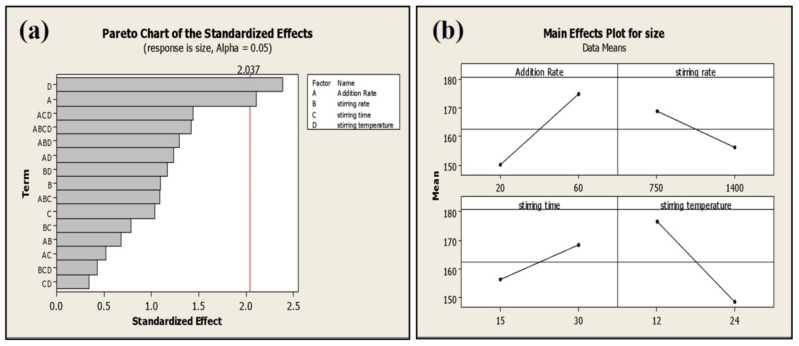
Pareto (**a**) and main effect plot (**b**) of process parameters (PP) on average particle size.

The addition rate and stirring temperature are the most significant (*p* < 0.05) factors affecting the average particle size of phytosomes. Batches processed at a high addition rate exhibited smaller particle size (135 ± 6 nm) compared to the batches processed at a low addition rate (159 ± 3 nm) (Figure 6). 

**Figure 7:**
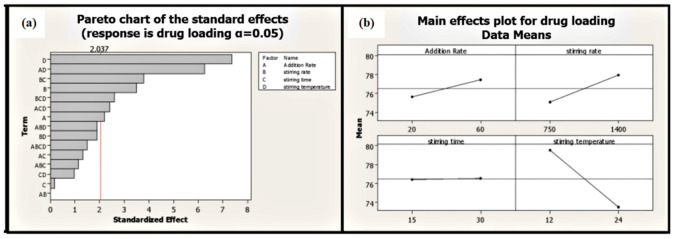
Pareto (**a**) and main effect plot (**b**) of process parameters (PP) on drug loading efficiency.

Stirring temperature perspective batches processed at high temperatures exhibited low average particle size compared to batches processed at low temperatures (Table 1). Moreover, stirring temperature is the most significant factor affecting EGCG loading on phospholipid (*p* ≤ 0.001). Phytosomal batch processed at low temperature led to high loading efficiency of up to 86.68% (Figure 7 and Table 1).

**Figure 8:**
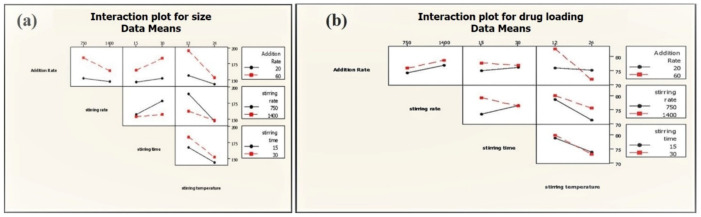
Interactions of PP on average particle size (**a**) and drug loading (**b**) of phytosome.

Two-way interactions between process parameters were not significant for the average particle size of phytosome, which was, however, found to have a significant impact on the drug loading of phytosome (addition rate: Stirring temperature; stirring speed: Stirring time) (Figure 8). Slow addition rate and low stirring temperature lead to high drug loading (86.68%) at high stirring speed (Table 2).

**Figure 9:**
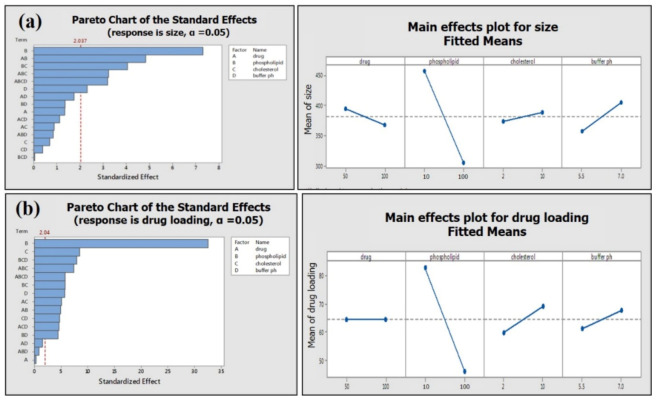
Pareto chart and main effect plot of material attributes (MA) on (**a**) average phytosome size and (**b**) drug loading efficiency.

**Figure 10:**
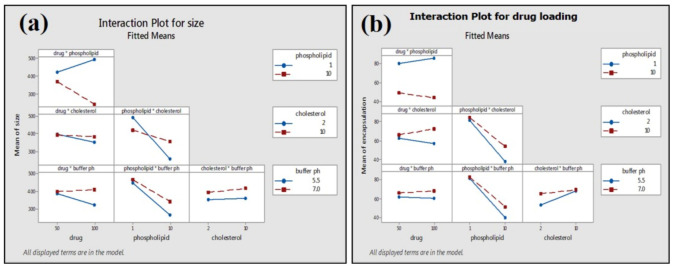
Interactions of MA on average particle size (**a**) and drug loading (**b**) of phytosome.

Phospholipid concentration showed the most significant (*p* ≤ 0.001) impact on the average particle size and drug loading on phospholipid (Figure 9). The high concentration of phospholipid results in small average particle size and less loading efficiency (Table 2; Figure 9a,b). Two-way interactions of material attributes (MA) are found to have a marked impact on average particle size and drug loading on phospholipid. However, two-way interactions of material attributes showed a greater effect on the drug loading efficiency of green tea extract compared to the average size of the phytosome (Figure 10).

### 2.7. Stability Testing

**Figure 11:**
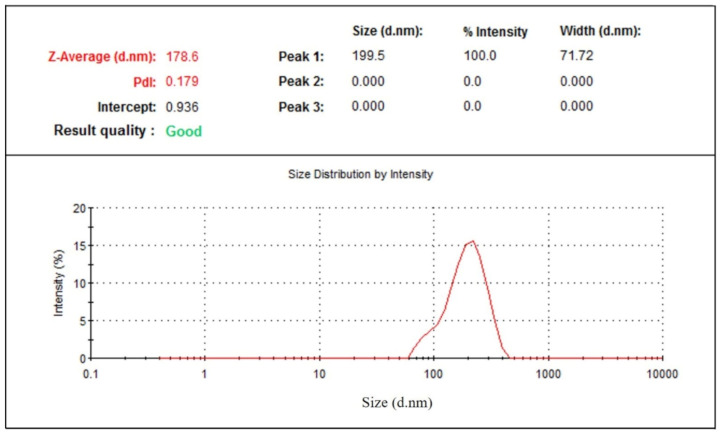
Particle size distribution of nanophytosome (batch 16) after one month.

The physical stability of prepared green tea extract-loaded nanophytosome was observed after one month at ambient conditions (25 ± 1 °C and 65 ± 5% RH). The results (Table 1) show that batch 16 with the highest drug loading (86.68%) has an average particle size of 173 ± 4 nm, which after one month exhibits an average particle size of 178 ± 6 nm (Figure 11) and drug loading of 84.07%. No significant changes in average particle size and drug loading were observed for the phytosomal preparation stored at 25 °C and 65% RH.

### 2.8. Anti-Inflammatory Study of Green Tea Extract and EGCG-Loaded Nanophytosome

**Figure 12:**
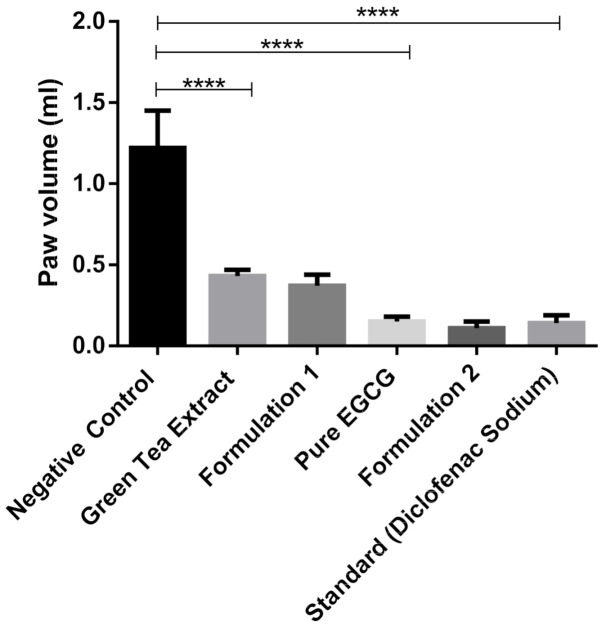
Anti-inflammatory study of green tea extract, pure EGCG and its phytosomal preparations after four days (mean value with 95% CI, where **** means *p* ≤ 0.0001).

Green tea extract, pure EGCG and its phytosomal preparations exhibited significant anti-inflammatory activity (*p* ≤ 0.001) against acute edema when compared to that of the negative control group (Figure 12 and Table 3). It was observed that the phytosomal preparation of pure EGCG showed the highest reduction in carrageenan-induced paw edema (Table 3), significantly higher (*p* ≤ 0.001) compared to the negative control group and higher than other groups used during the anti-inflammatory study.

This study suggests that green tea extract and pure EGCG have strong anti-inflammatory activity, which is also discussed by Bae et al. (2020) [38]. EGCG prevents inflammatory reactions induced by different types of cells, for example, immune cells, fibroblasts and vascular endothelial cells [39]. While phytosomal preparation of green tea extract and pure EGCG showed better anti-inflammatory activity compared to extract and pure EGCG (Figure 12), it was not significant. This phenomenon is probably due to the high solubility of EGCG in water; therefore, nanophytosomal preparations did not show significant improvement in anti-inflammatory activity

## 3. Materials and Methods

### 3.1. Materials

Green tea leaves used in this study were collected from a local tea garden in Bangladesh. Phospholipid used in this study was extracted in house from egg yolk [39]. All solvents used in this study were HPLC grade and were obtained from Sigma Aldrich, India. Sodium chloride and hydroquinone were obtained from the laboratory store of North South University. Cholesterol was purchased from Loba Chemie (Maharastra, India). Pure EGCG (Epigallacto catechin gallate) was purchased from sigma Aldrich, (Darmstard, Germany). 

### 3.2. Methods

#### 3.2.1. EGCG Extraction from Green Tea Leaves

The green tea leaves were rinsed and then air dried in a shady place at 25 °C for 10 days. The dried grounded green tea leaves along with distilled water were heated in a water bath at 80 °C for 40 min at 150 rpm. The extract was filtered and was equilibrated in an equal volume of chloroform to eliminate impurities. The catechin available in the extract was collected from the water layer using an equal volume of ethyl acetate. Then it was concentrated using a rotary evaporator and collected.

#### 3.2.2. Analysis of Catechin (EGCG) by UHPLC Systems

The sample was prepared by dissolving pure catechin (EGCG) powder in methanol. The study employed the Thermo Scientific Ultimate 3000^®^ UHPLC systems. Sample separation was carried out by reverse-phase isocratic elution in a mobile phase consisting of methanol, water and 0.25% formic acid (FA) (20/80% *v*/*v*) at a flow rate of 0.2 mL/min through an Acquity^®^ UHPLC column HSS C18 (2.1 × 50 mm, 1.7 μm). The column temperature was maintained at 35 °C for a total run time of 2.5 min. The catechin peak was eluted at 1.617 min. A freshly prepared mobile phase was filtered using a 0.22 µm filter paper. This was followed by continuous degassing in the UHPLC system by an online degasser. The detector wavelength was set at 200 nm with an injection volume of 1.0 µL.

#### 3.2.3. Analysis of Catechin (EGCG) by GC/MS

Gas Chromatography (Varian CP-3800) in combination with Mass Spectroscopy (Model-TQ-320 MS) was used for the analysis of EGCG. The ion source was at 250 °C and the inlet line temperature was set to 250 °C. The chromatographic column was VF-5MS column (30 m × 0.25 mm × 0.25 µm film thickness), with high-purity helium as the gas carrier at a flow rate of 1 mL/min. The temperature of the injector was at 280 °C and with a splitless injector at 20:1. The column was initially set at 40 °C and the temperature increased gradually to 150 at 20 °C/min and held for 1 min and then again increased to 300 °C with the rate of 20 °C/min and held for a further 1 min.

#### 3.2.4. Nanophytosomal Preparation Process of Epigallocatechin-3-Gallate (EGCG)

Nanophytosomal batches of EGCG were prepared by dissolving green tea extract or pure EGCG in 40 mL of phosphate buffer solutions (PBS) and stirred for 15 min. Phospholipids along with cholesterol, in a ratio of 1:5 (cholesterol: phospholipids), were dissolved in 10 mL of ethanol and stirred for 15 min. The PBS solution was then injected into an ethanol solution and mixed for 20 min with a magnetic stirrer. Ethanol was evaporated by stirring the mixture of solution at room temperature (25 °C). Phytosomal preparations were then stored in a glass bottle at room temperature. 

#### 3.2.5. Design of Experiment (DoE)

Design of experiment (DoE) was used to evaluate the impact of process parameters (PP) and material attributes (MA) on the quality attributes of phytosomal preparation of green tea extract. Each of the factors was studied at two levels (high = H and low = L), where preliminary studies were utilized to identify the parameter range for the detailed study.

A full factorial experimental design 2^4^ was used to understand the impact of process parameters—addition rate ((H = 20 s, 2 mL/s) (L = 60 s, 0.67 mL/s)), stirring speed (H = 1400, L = 700), stirring time (H = 30 min, L = 15 min) and stirring temperature (H = 24 °C, L = 12 °C)—on the average particle size and drug loading efficiency of phytosome (Table 4). The impact of material attributes—drug concentration (H = 100 mg, L = 50 mg), phospholipid concentration (H = 100 mg, L = 10 mg), cholesterol concentration (H = 10 mg, L = 2 mg) and buffer (H = pH 7.0 and L = pH 5.5)—was also investigated on the average particle size and drug loading efficiency of phytosome (Table 5).

#### 3.2.6. Particle Size Analysis of Phytosome

The particle size distribution of the phytosomal preparations of green tea extract and pure EGCG were characterized using Malvern Zetasizer Nano-ZS (Model ZEN3600, Malvern Instruments, Malvern, UK) at 25 °C. The analysis was performed in triplicate and the average value was calculated.

#### 3.2.7. Scanning Electron Microscopy (SEM)

The surface characteristic of the phytosomal preparations of green tea extract was visualized using a (Carl Zeiss AG, Jena, Germany) scanning electron microscope (SEM). The freeze-dried phytosomal nanosuspensions were put onto a double-sided adhesive carbon tape and analyzed at different magnifications. The samples were coated by Jeol JFC-1600 (Tokyo, Japan) auto fine coater for a few minutes before analysis. 

#### 3.2.8. Transmission Electron Microscopy (TEM)

The optimized phytosomal batches of green tea extract were characterized using a transmission electron microscopy Jeol JEM1010 (Tokyo, Japan) [40]. A vesicle was freshly prepared and diluted using distilled water. The sample was then placed on the carbon-coated copper grid to dry. Osmium was used to stain the lipid components and after drying it was loaded into the TEM and viewed at different magnifications.

#### 3.2.9. Stability Testing

A stability study of nanophytosomal batches of green tea extract was performed at 25 °C and 60% relative humidity (RH). Particle size distribution data at the initial stage and after 1 month were determined using Malvern Zetasizer.

#### 3.2.10. In Vivo Anti-Inflammatory Study of Green Tea Extract and EGCG-Loaded Nanophytosome

##### Animals

In this study, thirty-six long-Evans rats (24 female and 12 male) with an average weight of 190 g, obtained from North South University’s animal house were used. Animal experimentation procedures were performed according to the National Institute of Health Guide for the Care and Use of Laboratory Animals (NIH Publications No. 80–23; 1996, revised 1978) as well as institutional guideline established by the institutional animal care and use committee (IACUC), North South University, Dhaka, Bangladesh. The experimental procedure was reviewed and approved by the institutional animal ethics committee (project identification code- AEC-09-2018, approved on 04/08/2018) at North South University, Dhaka, Bangladesh [41].

##### Methods

Thirty-six rats (24 female and 12 male) were weighed and divided into six groups (4 female and 2 male in each group). The negative control group (Group 1) was treated with 0.1 mL of 1% solution of carrageenan in 0.9% saline. The extract group (Group 2) was treated with 0.1 mL of 1% solution of carrageenan and green tea extract (80 mg/kg body weight of rat). Formulation 1 group (Group 3) treated with 0.1 mL of 1% solution of carrageenan and phytosomal preparation of *green tea* extract (80 mg/kg body weight of rat). Formulation 2 group (Group 5) was treated with 0.1 mL of 1% solution of carrageenan and phytosomal preparation of pure EGCG (10 mg/kg body weight of rat). Pure EGCG group (Group 4) was treated with 0.1 mL of 1% solution of carrageenan and pure EGCG (10 mg/kg body weight of rat). The standard group (Group 6) was treated with 0.1 mL of 1% solution of carrageenan and diclofenac sodium (10 mg/kg body weight of rat) [41].

A period of one week was enough to allow the rats to adapt to the laboratory conditions. Plethysmometer was used to measure the displacement volume of all paws in the studied groups. 0.1 mL solution of 1% carrageenan solution (prepared using 0.9% saline) was injected subcutaneously into the sub-plantar region of the left hind paw of all the experiment rats. On the first day, after 5 h of the carrageenan administration, the paw volume of all rats was measured using a plethysmometer. Since the volume of drug suspension or formulation was large, in this study it was administered twice one after another. The first dose of drug or formulation was administered intraperitoneally (IP) for all six groups of rats except the negative control group, which was treated with 0.9% saline only. Similarly, the second dose of the drug was administered IP for all group of rats 10 min after the first dose. [41]. 

From the second day to the fourth day, negative control, extract, Formulations 1 and 2, pure EGCG and standard drug were administered in the same manner as mentioned before. The paw volume of all rats was measured using a plethysmometer after 3 h of the second dose of drug or formulation [41].

## 4. Conclusions

UHPLC and GCMS results suggest that catchins (EGCG) were present in green tea extract. UHPLC analysis showed that 500 μg EGCG was present per mL of green tea extract. Critical processing conditions and material attributes were identified through a designed study based on the philosophy of (QbD). The results confirmed that the different factors used in this study affect the average particle size and drug loading of phytosomes. This study suggests that the addition rate, stirring (mixing of two liquids) temperature and phospholipid concentration are the three most critical parameters affecting both average particle size and drug loading on phytosome. Drug loading is markedly dependent on the average size of the phytosome, with the large-size phytosome having high drug loading which was also observed for liposomal delivery in our previous study. Therefore, there is a need to work or design on the question of whether we will prefer liposome or phytosome with an average particle size of 100–150 nm with ≥80% drug loading efficiency or average particle size < 50 nm with ~20% drug loading efficiency. Results demonstrated that pure EGCG and its phytosomal preparation have an anti-inflammatory activity which showed significant inhibition of carrageenan-induced rat paw edema. Stability studies showed that the phytosomal preparation was stable when stored at ambient conditions for one month. 

## Figures and Tables

**Table 1:** Design of Experiment (DoE) of process parameters (PP) for nanophytosome batches. H and L denote high and low, respectively.

Batch	Addition Rate	Stirring Speed	Stirring Time	Stirring Temperature	Particle Size (nm) ± SD	Drug Loading Efficiency (%)
1	H	H	H	H	146 ± 3	74.25
2	H	H	H	L	159 ± 4	77.02
3	H	H	L	L	150 ± 7	77.97
4	H	L	L	L	170 ± 10	70.18
5	L	L	L	L	180 ± 16	81.46
6	L	L	L	H	151 ± 5	63.29
7	L	L	H	H	148 ± 2	68.27
8	L	H	H	H	156 ± 5	75.51
9	H	L	H	H	144 ± 8	74.50
10	H	L	H	L	147 ± 8	79.08
11	H	L	L	H	133 ± 5	72.96
12	L	H	L	H	159 ± 3	74.10
13	L	H	H	L	169 ± 2	81.49
14	L	L	H	L	262 ± 28	84.80
15	H	H	L	H	135 ± 6	78.93
16	L	H	L	L	173 ± 4	86.68

**Table 2:** Design of Experiment (DoE) of material attributes (MA) for nanophytosome batches. H and L denote high and low, respectively.

Batch	Drug conc.	Phospholipid conc.	Cholesterol conc.	Buffer	Particle Size (nm)	Drug Loading Efficiency (%)
1	H	H	H	H	256 ± 52	48.47
2	H	H	H	L	430 ± 3	80.25
3	H	H	L	L	560 ± 40	86.93
4	H	L	L	L	547 ± 47	84.23
5	L	L	L	L	358 ± 28	52.59
6	L	L	L	H	310 ± 21	64.59
7	L	L	H	H	479 ± 36	89.64
8	L	H	H	H	461 ± 25	91.70
9	H	L	H	H	394 ± 20	84.37
10	H	L	H	L	428 ± 31	81.55
11	H	L	L	H	436 ± 26	52.84
12	L	H	L	H	316 ± 19	46.59
13	L	H	H	L	326 ± 27	53.34
14	L	L	H	L	449 ± 21	76.42
15	H	H	L	H	400 ± 42	41.96
16	L	H	L	L	414 ± 35	78.02

**Table 3:** Anti-inflammatory study of green tea extract, pure EGCG and its phytosomal preparations.

Time	Negative Control	EGCG Extract		Formulation 1		EGCG Pure		Formulation 2		Standard	
	Paw Volume (mL)	Paw Volume (mL)	(%) Reduction	Paw Volume (mL)	(%) Reduction	Paw Volume (mL)	(%) Reduction	Paw Volume (mL)	(%) Reduction	Paw Volume (mL)	(%) Reduction
3h (1 day)	1.20 ± 0.47	0.90 ± 0.23	25	1.16 ± 0.41	3.33	0.36 ± 0.15	70	0.41 ± 0.18	65.83	0.57 ± 0.07	52.5
5h (1 day)	1.97 ± 0.15	1.44 ± 0.14	26.9	1.84 ± 0.11	6.6	0.62 ± 0.26	68.53	0.46 ± 0.16	76.65	0.77 ± 0.08	60.91
2 days	1.76 ± 0.32	1.08 ± 0.11	38.64	1.39 ± 0.12	21.02	0.30 ± 0.15	82.95	0.21 ± 0.14	88.07	0.36 ± 0.10	79.55
3 days	1.53 ± 0.23	1.06 ± 0.23	30.72	0.89 ± 0.30	41.83	0.20 ± 0.13	86.93	0.14 ± 0.14	90.85	0.19 ± 0.04	87.58
4 days	1.10 ± 0.21	0.39 ± 0.13	64.55	0.37 ± 0.15	66.8	0.14 ± 0.12	87.27	0.10 ± 0.13	88.18	0.13 ± 0.05	88.18

**Table 4:** Design of experiment (DoE) of process parameters for phytosome preparation. H and L denote high and low, respectively.

Batch	1	2	3	4	5	6	7	8	9	10	11	12	13	14	15	16
Addition rate	H	H	H	H	L	L	L	L	H	H	H	L	L	L	H	L
Stirring speed	H	H	H	L	L	L	L	H	L	L	L	H	H	L	H	H
Stirring time	H	H	L	L	L	L	H	H	H	H	L	L	H	H	L	L
Stirring temperature	H	L	L	L	L	H	H	H	H	L	H	H	L	L	H	L

**Table 5:** Design of experiment (DoE) of material attributes (MA) for phytosome preparation. H and L denote high and low, respectively.

Batch	1	2	3	4	5	6	7	8	9	10	11	12	13	14	15	16
Drug Concentration	H	H	H	H	L	L	L	L	H	H	H	L	L	L	H	L
Phospholipid Concentration	H	H	H	L	L	L	L	H	L	L	L	H	H	L	H	H
Cholesterol Concentration	H	H	L	L	L	L	H	H	H	H	L	L	H	H	L	L
Buffer	H	L	L	L	L	H	H	H	H	L	H	H	L	L	H	L
